# Defining the Role of Dexmedetomidine in the Prevention of Delirium in the Intensive Care Unit

**DOI:** 10.1155/2015/635737

**Published:** 2015-10-19

**Authors:** S. Nelson, A. J. Muzyk, M. H. Bucklin, S. Brudney, J. P. Gagliardi

**Affiliations:** ^1^Critical Care, Hospital Pharmacy Services, Mayo Clinic, Rochester, MN 55905, USA; ^2^Department of Pharmacy Practice, Campbell University School of Pharmacy and Health Sciences, Buies Creek, NC 27506, USA; ^3^Department of Psychiatry & Behavioral Sciences, Duke University, Durham, NC 27710, USA; ^4^Department of Clinical Pharmacy, University of Tennessee Medical Center, Knoxville, TN 37920, USA; ^5^Departments of Anesthesiology and Medicine, Duke University Medical Center, Durham VAMC, Durham, NC 27710, USA; ^6^Department of Medicine, Duke University, Durham, NC 27710, USA

## Abstract

Dexmedetomidine is a highly selective *α*_2_ agonist used as a sedative agent. It also provides anxiolysis and sympatholysis without significant respiratory compromise or delirium. We conducted a systematic review to examine whether sedation of patients in the intensive care unit (ICU) with dexmedetomidine was associated with a lower incidence of delirium as compared to other nondexmedetomidine sedation strategies. A search of PUBMED, EMBASE, and the Cochrane Database of Systematic Reviews yielded only three trials from 1966 through April 2015 that met our predefined inclusion criteria and assessed dexmedetomidine and outcomes of delirium as their primary endpoint. The studies varied in regard to population, comparator sedation regimen, delirium outcome measure, and dexmedetomidine dosing. All trials are limited by design issues that limit our ability definitively to conclude that dexmedetomidine prevents delirium. Evidence does suggest that dexmedetomidine may allow for avoidance of deep sedation and use of benzodiazepines, factors both observed to increase the risk for developing delirium. Our assessment of currently published literature highlights the need for ongoing research to better delineate the role of dexmedetomidine for delirium prevention.

## 1. Introduction

Delirium is an acute fluctuation in mental status that manifests with inattention, disorganized thinking, and/or an altered level of consciousness [1]. It occurs often in critically ill patients, with as many as 20% to 50% of nonventilated patients and 60% to 80% of mechanically ventilated patients reported to develop delirium [1]. Intensive care unit (ICU) delirium is associated with a number of detrimental outcomes including longer ICU and hospital length of stay, increased costs, and long-term cognitive impairment [2–5]. Additionally, patients who develop ICU delirium experience increased mortality risk, with rates exceeding three times those of matched, nondelirious ICU patients within 6 months of critical illness [2]. A number of modifiable and nonmodifiable risk factors may predispose patients to ICU delirium [6]. Medications used for sedation, agitation, and anxiety are widely considered the most influential in terms of ICU delirium risk. Current strategies for delirium prevention focus on maximizing nonpharmacologic interventions, effectively treating pain, allowing for early mobility of patients and implementation of sedation protocols targeting light sedation, often including daily sedation interruption and the avoidance of benzodiazepines [6–8].

Benzodiazepines are historically the medication class of choice for provision of sedation and treatment of agitation in the ICU; however, they repeatedly have been identified as a risk factor for development of delirium in adult ICU patients [8–10]. As the presence of delirium has been associated with negative outcomes, delirium prevention may be the most effective way to reduce its associated morbidity and mortality [11]. The identification of a nondeliriogenic sedation strategy is therefore of paramount importance for critical care medicine. Roberts and colleagues emphasize this point and conclude that a shift in practice is needed in considering the pharmacology of sedatives and the duration of sedation [12].

Currently, no medications are approved by the Food and Drug Administration (FDA) for the prevention of ICU delirium. Guidelines for the treatment of pain, agitation, and delirium in critically ill patients supported by the Society of Critical Care Medicine also do not provide recommendations on pharmacologic delirium prevention [8]. However, many studies have suggested that the use of dexmedetomidine for sedation is associated with lower incidence of delirium and improved outcomes associated with delirium when compared to benzodiazepine or opioid-based sedation strategies in mechanically ventilated patients; dexmedetomidine also has been associated with lower rates of agitation in delirious patients compared to conventional treatments [13–19].

We present a systematic review of randomized controlled trials (RCTs) reporting on the efficacy of dexmedetomidine with respect to a primary outcome of delirium prevention in any ICU population. We surmise that adult patients receiving sedation with dexmedetomidine as compared to nondexmedetomidine sedation strategies will exhibit a lower incidence of delirium.

## 2. Literature Identification

A search was performed of MEDLINE, EMBASE, and the Cochrane Database of Systematic Reviews from 1966 through April 2015. Search terms included “dexmedetomidine,” “delirium (MeSH term),” “ICU psychosis,” “encephalopathy,” “alpha-2 agonist,” and “agitation.” The search was limited to the English language and human subjects. Study type was limited to randomized controlled trials (RCTs), controlled trials, or comparative studies. Reference lists of articles retrieved were searched for the identification of any possibly relevant publications. The search was last performed on 20 April 2015 independently by two authors (S. Nelson and A. J. Muzyk).

Studies were eligible for inclusion if they were RCTs of adult subjects (18 years or older) hospitalized in any ICU and were designed to assess the primary outcome of dexmedetomidine's efficacy on delirium prevention in a prospective manner. To be included in the systematic review studies were required to employ validated delirium detection or diagnostic tools such as the Confusion Assessment Method for the ICU (CAM-ICU), the Intensive Care Delirium Screening Checklist (ICDSC), or the Diagnostic and Statistical Manual of Mental Disorders (DSM-IV-TR) definition in conjunction with the Delirium Rating Scale (DRS) [20–22]. Nonrandomized trials and those that report solely on delirium measures as secondary outcomes were excluded but were retained for discussion and context.

Two authors (A. J. Muzyk and J. P. Gagliardi) read abstracts independently and assessed appropriateness for inclusion in the systematic review. Disagreements were resolved by discussion with a third author (M. Bucklin) until consensus was reached. For each study, data necessary for critical evaluation were extracted (see Table 1). This includes study design, inclusion and exclusion criteria, patient population and demographic data, intervention (including drug and dosing utilized), method of delirium detection, and results. Articles were appraised for validity using the Jadad [23] score (which ranges from 0: very poor to 5: rigorous).

Seventy-one studies were identified through initial literature search, with 42 abstracts meeting inclusion requirements; 17 articles from MEDLINE, 21 articles from EMBASE, and four articles were found in the Cochrane Database of Systematic Reviews. Of these, 38 were excluded because they were case reports, nonrandomized open-label trials, or review articles, or they did not report on delirium as the primary outcome. No additional articles were identified based on reference review. This left 4 RCTs of dexmedetomidine for the prevention of delirium. One study was excluded as it was a subgroup analysis of data reported in another of the eligible trials.

The three remaining trials are summarized below [13–15]. As they differ significantly in regard to baseline patient characteristics, ICU type, primary outcome, the use of an active comparator, and dosing of dexmedetomidine, a meta-analysis was not appropriate and not undertaken.

## 3. Literature Evaluation

The first RCT was conducted by Pandharipande and colleagues [13]. They included adults hospitalized at two tertiary care hospitals in medical and surgical ICUs who received mechanical ventilation for at least 24 hours. Exclusion criteria targeted potential subjects in whom delirium assessment would be difficult (non-English speaking or severe hearing difficulties) or confounded (neurological disease, severe dementia, and Child-Pugh class B or C) and in whom benzodiazepine therapy may be necessary (active seizure, alcohol abuse, and benzodiazepine dependency). The exclusion criteria also targeted those at risk of cardiotoxicity of dexmedetomidine (active myocardial infarction and second- or third-degree heart block) and those who were pregnant or lactating. Potential subjects were also excluded if family or physician refused consent or if they were moribund, with planned withdrawal of life support. Subjects were randomized to a double-blind infusion of either dexmedetomidine (dose ranges from 0.15 to 1.5 mcg/kg/h) or lorazepam (dose ranges from 1 mg/h to 10 mg/h). Presence of delirium was assessed for 12 days or until hospital discharge using the CAM-ICU, and coma was assessed utilizing the Richmond Agitation Sedation Scale (RASS), with coma defined as a RASS score of −4 or −5. Primary outcomes of this study were a composite end point of delirium-free and coma-free days and the percentage of days spent within 1 RASS point of the predefined sedation goal.

One hundred and three subjects were included in the primary analysis with no significant differences in baseline characteristics between treatment groups, though a higher proportion of subjects randomized to dexmedetomidine had received fentanyl prior to randomization. The majority of subjects were hospitalized for sepsis, acute respiratory distress syndrome, or for a pulmonary complication. Compared to subjects randomized to lorazepam, those sedated with dexmedetomidine experienced more days without coma or delirium (7 days dexmedetomidine versus 3 days lorazepam, *p* = 0.01) and spent more time within 1 point of their RASS goal (67% dexmedetomidine versus 55% lorazepam, *p* = 0.008). Additionally, significantly fewer subjects treated with dexmedetomidine experienced any delirium or coma compared to those treated with lorazepam. There was no significant difference between treatment groups on secondary outcomes of delirium-free days, duration of delirium, or prevalence of delirium. There was also no difference observed in terms of concomitant medications used for sedation or delirium, mechanical ventilator free days, ICU length of stay, 28-day mortality, or 12-month mortality. Regarding safety, subjects randomized to dexmedetomidine had significantly more sinus bradycardia (17% versus 4%, *p* = 0.03); one subject in each group had a heart rate less than 40 beats per minute. The rate of self-extubation did not differ between groups.

Benefits of dexmedetomidine over lorazepam were demonstrated only in the composite endpoints. Individual endpoint analysis yielded a statistically significant benefit of dexmedetomidine on rate of coma but no difference between sedation strategies in terms of delirium prevalence (79% dexmedetomidine versus 82% lorazepam, *p* = 0.65) and delirium-free days (9 dexmedetomidine versus 7 lorazepam, *p* = 0.09). Similarly, the use of antipsychotics, presumably for the treatment of delirium, was not significantly different between groups (46% dexmedetomidine versus 35% lorazepam, *p* = 0.26), further suggesting that the extent of delirium was not reduced with the use of dexmedetomidine. As noted above, the study may have been biased in that more subjects randomized to dexmedetomidine had received fentanyl prior to assignment to a study protocol. It is possible that the prior use of opioids (which may predispose subjects to delirium) contributed to the development of delirium in the dexmedetomidine group. While these limitations are significant, it is important to consider the ability of dexmedetomidine to achieve the goal of light sedation, a concept which is becoming increasingly important for the reduction of delirium in all critically ill patients. Also, it is unclear how often the CAM-ICU was implemented to screen for delirium. As delirium is a waxing and waning state, more rigorous CAM-ICU assessments may have identified more delirium, affecting the outcome observed.

The second RCT conducted by Shehabi and colleagues included adults aged 60 years or greater undergoing on-pump cardiac surgery at one of two tertiary care hospitals [14]. Potential subjects were excluded if they reported an allergy to any study medication, had baseline dementia, Parkinson's disease, or a seizure disorder, were receiving other *α*_2_ agonists, or were on psychoactive medications, excluding nighttime hypnotics. Additionally, subjects were required to understand the English language, to have had a preoperative heart rate > 55 bpm and/or systolic blood pressure >90 mmHg, to weigh ≤150 kg, and to have no significant renal impairment (serum creatinine ≤1.6 mg/dL or creatinine clearance ≥50 mL/min). Subjects were randomized to a blinded infusion of either dexmedetomidine (dose ranges from 0.1 mcg/kg/h to 0.7 mcg/kg/h) or morphine (10 mcg/kg/h to 70 mcg/kg/h) without the use of loading doses. Infusions were titrated based on a sedation protocol to a goal Motor Activity Assessment Scale (MAAS) of 2 to 4. Open-label propofol (bolus and/or infusion) was allowed in both groups for hypertensive episodes and unplanned awakening or to meet goal sedation levels. Additionally, open-label morphine was administered as a 1 to 2 mg IV bolus dose given every 15 minutes to achieve adequate pain control in both groups. The primary outcome of this study was the percentage of subjects who developed delirium as measured using the CAM-ICU during the first five days following surgery.

Two hundred and ninety-nine subjects were included in the primary analysis, with 152 patients randomized to dexmedetomidine and 147 to morphine. There were no significant differences in baseline characteristic between the two groups. The majority of subjects were males, over the age of 65 years, hospitalized urgently for either a coronary bypass graft or a valve replacement with/without a coronary graft requiring a cross clamp time of approximately 60 minutes. Authors found the incidence of delirium within five days of surgery not different between treatment groups (8.5% in dexmedetomidine versus 15% in morphine, *p* = 0.088). A secondary outcome of delirium duration was shorter for subjects randomized to dexmedetomidine versus morphine sedation (2 days versus 5 days, resp., *p* = 0.0317). Subgroup analysis of those requiring intraaortic balloon pump (IABP) for hemodynamic support demonstrated a lower incidence of delirium in the dexmedetomidine group compared with the morphine group (15% versus 36%, resp., *p* = 0.001). Propofol was required in more than 75% of all subjects regardless of treatment assignment within 6 hours of cardiac surgery and was required in more than one-third of all subjects thereafter. Morphine was used with similar rates between study groups (approximately 12% of all subjects 6 hours postoperatively). In regard to safety, subjects randomized to dexmedetomidine experienced significantly more bradycardia (16.5% versus 6.1%, *p* = 0.006) and significantly less systolic hypotension (23% versus 38.1%, *p* = 0.006).

Several limitations of this trial warrant discussion. As with any study that aims to distinguish between sedation and delirium, it may have been challenging to identify the optimal timing for delirium assessment, and given the sedation of subjects included in the study, delirium may have been underrecognized in both groups. Second, the majority of subjects received propofol at some point during the postoperative period, which clouds the purity of the comparison between dexmedetomidine and morphine sedation strategies. Finally, while investigators used a standard sedation protocol familiar at the study sites, utilization of the MAAS is not commonplace in the United States, making it more difficult to accurately replicate or generalize the study methodology.

The final study meeting inclusion criteria was conducted by Maldonado and colleagues [15]. They compared the incidence of postoperative delirium in an open-label, randomized, controlled fashion in patients undergoing cardiac surgery randomized to dexmedetomidine, propofol, or midazolam. Subjects were included if they were undergoing elective valve operations and between the ages of 18 and 89. Exclusion criteria included preexisting dementia or schizophrenia, preoperative psychotropic medication use, active or recent substance abuse/dependence, stroke within the last six months, advanced heart block, pregnancy, or anticipated need for deep hypothermic circulatory arrest. Subjects were randomized to an infusion of dexmedetomidine (loading dose 0.4 mcg/kg and maintenance dose ranging from 0.2 mcg/kg/h to 0.7 mcg/kg/h), propofol (dose ranges from 25 mcg/kg/min to 50 mcg/kg/min), or midazolam (dose ranges from 0.5 mg/h to 2 mg/h). Subjects received no other agents to achieve or maintain goal sedation level (Ramsay score of 3 for intubated patients and 2 for nonintubated patients). Delirium was diagnosed based on DSM-IV-TR criteria and confirmed using the Delirium Rating Scale (DRS).

All subjects could receive fentanyl 25 to 50 mcg intravenously every hour as needed for pain within the first 24 hours postoperatively and ketorolac, hydrocodone, or oxycodone thereafter. If delirium was suspected, subjects could receive haloperidol up to 5 mg intravenously every 6 hours as needed in the first 24 hours, with haloperidol or lorazepam as necessary thereafter. The primary outcome was the proportion of patients in each treatment group diagnosed with postoperative delirium within the first 3 days following cardiac surgery.

A total of 118 subjects met inclusion and exclusion criteria and were randomized to one of three treatment arms. Authors report a 24% dropout rate among subjects, mostly due to protocol violation or clinical condition. Ninety subjects were included in the perprotocol analysis with all 118 included in the intention-to-treat analysis. Using both methods, subjects randomized to dexmedetomidine exhibited significantly less delirium than those randomized to propofol or midazolam (10% dexmedetomidine, 44% propofol, and 44% midazolam in intention-to-treat analysis, *p* < 0.001). Those randomized to dexmedetomidine also had fewer days of delirium (2 patient days dexmedetomidine, 45 patient days propofol, and 75 patient days midazolam, *p* < 0.001), though the duration of delirium was not significantly different between groups. Adjunctive fentanyl use was significantly higher in subjects randomized to midazolam (320 mcg dexmedetomidine, 364 mcg propofol, and 1088 mcg midazolam). Importantly, subjects randomized to propofol or midazolam were given more as-needed lorazepam and haloperidol for delirium. Though statistically nonsignificant, the percentage of subjects administered lorazepam was different between groups (3% dexmedetomidine, 23% propofol, and 20% midazolam, *p* = NS).

Strengths of this study include the use of protocolized anesthesia and postoperative care in all groups, application of a sedation protocol with a well validated sedation scoring system, and the selection of a homogenous patient population. Limitations in this study are numerous, however, including the use of the DSM-IV-TR to diagnose ICU delirium via a dedicated neuropsychiatrist and once daily delirium assessments. Additionally, this study did not report any data regarding the safety of the interventions evaluated. However the most notable limitations in this study are its open-label design, small sample size, and high dropout rate. Though statistically nonsignificant, the differential use of as-needed benzodiazepines may account for differences in delirium between treatment groups and may have nothing to do with the effect of dexmedetomidine itself.

## 4. Discussion

Delirium is associated with a number of deleterious outcomes; therefore, the prevention of its development is important. Dexmedetomidine is a highly selective *α*_2_ agonist that reduces norepinephrine release, producing sedation, anxiolysis, and sympatholysis. Numerous pathophysiologic mechanisms have been identified that may contribute to delirium development, and dexmedetomidine may be helpful for many of these [24, 25]. Excessive norepinephrine release, perhaps in response to infection or inflammation in critically ill adults, is part of a cascade of events hypothesized to lead to neuronal damage and the development of delirium [26]. Reduction in this neurotransmitter release through dexmedetomidine administration may prevent or limit the risk for delirium development. Excitatory amino acid (glutamate) toxicity, gamma-aminobutyric acid (GABA) agonism, and acetylcholine deficiency have also been implicated in the development of delirium [26, 27]. Dexmedetomidine therefore may also be beneficial for delirium prevention through decreasing glutamate release and its lack of GABA receptor modulation or cholinergic receptor activity when compared to other commonly used sedatives [28, 29]. Additionally, dexmedetomidine may promote natural sleep patterns through stimulation of *α*_2_ receptors leading to inhibition of noradrenergic neurons in the locus ceruleus and disinhibition of GABA neurons in the ventrolateral preoptic nucleus [30].

Despite these theoretical advantages, the evaluation of dexmedetomidine for the prevention of delirium in clinical trials has not clearly demonstrated benefit. The available body of literature addressing this topic as a primary endpoint is limited in quantity and quality. Other studies investigating the benefit of dexmedetomidine have been reviewed in four meta-analyses [31–34]. These meta-analyses are unique from our review as they allowed for assessment of various delirium outcomes, included delirium outcomes as primary and secondary endpoints, were often focused on select patient populations, and concurrently evaluated the effect of other interventions on the outcome of delirium. Given the heterogeneity of the previously published analyses, outcomes regarding the influence of dexmedetomidine on delirium in these studies are varied. Zhang and colleagues published a meta-analysis which demonstrated a lower rate of delirium in postoperative patients receiving dexmedetomidine [31]. These findings differed from an earlier meta-analysis demonstrating no significant reduction in delirium with dexmedetomidine in a general population of critically ill adults in the ICU [32]. Differences in methodology, populations, and measurements make a comparison between meta-analyses difficult, though one possibility is that dexmedetomidine may be more effective in preventing delirium among surgical, but not medical, patients [25, 33, 34].

In our review of the available literature examining prevention of delirium as a primary endpoint, utilization of dexmedetomidine did not routinely demonstrate benefit among all ICU patient populations. This review is limited by the small number of trials that met robust inclusion criteria, use of varied comparator agents, and the inclusion of trials that inherently had their own limitations or bias, including open-label studies.

Despite methodological limitations or the exclusivity of critically ill populations examined, a common concept that can be extracted from available literature is the benzodiazepine-sparing benefit of dexmedetomidine. Benzodiazepines are a known risk factor for the development of delirium and their use has been associated with oversedation and prolonged mechanical ventilation. Utilization of dexmedetomidine rather than benzodiazepines may afford the ability to maintain light sedation goals and an overall reduction in the risk for the development of delirium, providing a role for dexmedetomidine for the prevention of ICU delirium. The 2013 clinical practice guidelines for the management of pain, agitation, and delirium in adult ICU patients do not clearly delineate when dexmedetomidine should be utilized [8]. They do, however, suggest the use of nonbenzodiazepine sedation regimens, including dexmedetomidine, that allow for targeting light sedation. They also suggest that dexmedetomidine may be less deliriogenic as compared to other commonly used sedatives [8]. Additionally, dexmedetomidine possesses a favorable pharmacokinetic profile for use in critically ill adults. It has a short onset of action allowing for rapid sedation and ease of titration, an elimination half-life of two hours, minimal drug-drug interaction potential, and complete elimination from the body within 10 hours following infusion cessation [35]. Dexmedetomidine is rather well tolerated with most adverse effects being cardiac in nature. Bradycardia and hypotension are most common and usually occur with bolus and loading dose administration [36].

As interest grows in the area of delirium prevention, the role of dexmedetomidine should continue to be assessed and defined. It is important to note that other pharmacologic agents have been studied for delirium prevention, but demonstrating efficacy for delirium prevention has been elusive [37]. As no clear pharmacologic prevention strategy has been identified, use of dexmedetomidine to provide light sedation and avoid deliriogenic sedatives may be a reasonable therapeutic option until further information is available.

## 5. Conclusion

Dexmedetomidine is a centrally acting *α*_2_ agonist that is FDA-indicated for sedation in intubated, critically ill adults. It may allow for better attainment of light sedation goals, a strategy that is recommended in guidelines provided by the Society of Critical Care Medicine, rather than conventional sedation agents including benzodiazepines and opioids. Through attainment of light sedation and the sparing-effect of these other sedatives, dexmedetomidine may indirectly decrease the incidence of delirium. Its direct effect on the prevention of delirium remains theoretical and unproven. Continued attempts should be made to evaluate dexmedetomidine for this indication through larger, randomized, placebo controlled trials in varied critically ill patient populations.

## Figures and Tables

**Table 1:** Description of study methods and validity criteria.

	Pandharipande et al. (2007) [13]	Shehabi et al. (2009) [14]	Maldonado et al. (2009) [15]
ICU type	Medical/surgical	Cardiothoracic	Cardiothoracic
Subjects			
Number	103	299	90
Age (median, IQR)	Dexmedetomidine: 60 (49–65) Lorazepam: 59 (45–67)	Dexmedetomidine: 71.5 (66–76) Morphine: 71 (65–75)	Dexmedetomidine: 55 (16)^*∗*^ Propofol: 58 (18)^*∗*^ Midazolam: 60 (16)^*∗*^
Male gender (%)	51.5	75.3	63.6
Intervention (median, IQR)	Dexmedetomidine: 0.74 mcg/kg/h (0.39–1.04) Lorazepam: 3 mg/h (2.2–6)	Dexmedetomidine: 0.48 mcg/kg/h (0.23–0.76) Morphine: 49 mcg/kg/h (20–81)	Dexmedetomidine: 0.35 mcg/kg/h^*∗∗*^ Propofol: 26.3 mcg/kg/h^*∗∗*^ Midazolam: 1.5 mg/h^*∗∗*^
Primary outcome	Composite of delirium-free and coma-free days	Delirium incidence within 5 days of surgery	Incidence of post-operative delirium
Randomization	Computer generated, permuted blocks	Computer-generated blocks of 10	Random drawing the evening before surgery, blocked
Concealment	Known only to study pharmacist	Known only to study pharmacist	Open-label
Blinding	Solutions identical in color (clear)	Solutions identical in color (clear)	Unblinded
Jadad Score	4	3	2

*∗*: mean (SD); *∗∗*: mean.
